# The temporal expression patterns of brain transcriptome during chicken development and ageing

**DOI:** 10.1186/s12864-018-5301-x

**Published:** 2018-12-13

**Authors:** Zhongxian Xu, Tiandong Che, Feng Li, Kai Tian, Qing Zhu, Shailendra Kumar Mishra, Yifei Dai, Mingzhou Li, Diyan Li

**Affiliations:** 1 Institute of Animal Genetics and Breeding, College of Animal Science and Technology, Sichuan Agricultural University, Chengdu, 611130 Sichuan China; 2 Key Laboratory of Southwest China Wildlife Resources Conservation (Ministry of Education), China West Normal University, Nanchong, 637009 Sichuan China; 3 Novogene Bioinformatics Institute, Beijing, 100083 China

**Keywords:** Transcriptome, Chicken brain, Temporal expression patterns, Development and ageing

## Abstract

**Background:**

The transcriptional profiles of mammals during brain development and ageing have been characterized. However the global expression patterns of transcriptome in the chicken brain have not been explored. Here, we systematically investigated the temporal expression profiles of lncRNAs and mRNAs across 8 stages (including 3 embryonic stages, 2 growth stages and 3 adult stages) in the female chicken cerebrum.

**Results:**

We identified 39,907 putative lncRNAs and 14,558 mRNAs, investigated the temporal expression patterns by tracking a set of age-dependent genes and predicted potential biological functions of lncRNAs based on co-expression network. The results showed that genes with functions in development, synapses and axons exhibited a progressive decay; genes related to immune response were up-regulated with age.

**Conclusions:**

These results may reflect changes in the regulation of transcriptional networks and provide non-coding RNA gene candidates for further studies and would contribute to a comprehensive understanding of the molecular mechanisms of chicken development and may provide insights or deeper understanding regarding the regulatory mechanisms of age-dependent protein coding and non-protein coding genes in chicken. In addition, as the chicken is an important model organism bridging the evolutionary gap between mammals and other vertebrates, these high resolution data may provide a novel evidence to improve our comprehensive understanding of the brain transcriptome during vertebrate evolution.

**Electronic supplementary material:**

The online version of this article (10.1186/s12864-018-5301-x) contains supplementary material, which is available to authorized users.

## Background

Large-scale genomic studies have revealed that the majority of cellular RNAs are transcribed as non-protein coding RNAs (ncRNAs) [1–4]. Long non-coding RNAs (lncRNAs) are a subgroup of RNAs longer than 200 nucleotides (nt); many of them are 5′ capped, alternatively spliced and polyadenylated as mRNAs are [5]. LncRNAs are newly recognized regulating molecules and can be classified as intergenic lncRNAs (lincRNAs), intronic lncRNAs, antisense lncRNAs or sense-overlapping lncRNAs based on their genomic location [6]. Albeit mRNA-like lncRNAs have limited protein-coding potential, they function in many biological processes and play roles as signaling molecules, scaffolds, guides and decoys [7]. Transcriptional dynamics has been suggested to be a major contributor for brain structure development, as well as to the ageing [8]. Most lncRNAs are less conserved and expressed at significantly lower levels, as well as in more restricted temporal expression patterns, than mRNAs are, indicating that lncRNAs are of considerable importance in modulating various biological functions, especially development and cellular differentiation [9–11].

A broad range of transcriptional profiles in fish [9, 12], mouse [10, 13–16], monkeys [17–19] and humans [1, 20–26] during brain development and ageing have been reported in recent years. It has been demonstrated that the encoded information underlying development lies in regulatory elements that define different gene expression programs, which means the regulatory factors scale quadratically with genome size, the untranslated regions (UTRs) in mRNAs increase with the developmental complexity of organisms, and the non-protein coding intronic and intergenic transcripts also contain an expanded regulatory framework to control gene expression during differentiation and development [20]. Although remarkable achievements have been made in characterizing the transcriptional underpinnings of brain development and ageing in vertebrates, the precise mechanisms of lncRNAs in defining the complexity of brain functions remain unclear, particularly in birds. Although the chicken is now one of the most versatile experimental systems available, as an established model organism for the study of vertebrate development, the chicken is under evaluated in the study of developmental transcriptomics [27].

To systematically investigate the expression profiles of lncRNA and mRNA transcriptome in different stages, we performed high-throughput RNA sequencing to interrogate their temporal expression patterns in the chicken cerebrum. The present study provided an initial lncRNA catalogue of the chicken cerebrum, which improves our understanding of the temporal expression profile during development and ageing, and may provide insights or deeper understanding regarding the regulatory mechanisms of age-dependent genes in chicken. In addition, this lncRNA catalogue helps bridge the evolutionary gap between mammals and other vertebrates.

## Results

### Global identification of lncRNAs in the chicken cerebrum

To systematically investigate lncRNAs and their temporal expression profiles during development and ageing in the chicken cerebrum, a total of 21 cDNA libraries from 21 female Tibetan chickens at 8 life stages were constructed. The sampling times broadly spanned the life of the farm-raised chicken, representing 3 embryonic periods (embryonic days 12, 16, and 20: E12, E16 and E20), 2 growth periods (100 and 300 days of age: D100 and D300), and 3 adult periods (from young adults to ageing adults--1, 3, and 5 years of age: Y1, Y3 and Y5). Illumina sequencing yielded a total of 2,675,315,228 raw reads and 406.56 Gb of raw data with 150-bp paired-end RNA-Seq, 2,615,649,932 clean reads were obtained after quality control, corresponding to an average of 18.91 Gb of high-quality data per sample. Among them, 91.87–94.61% were mapped to the chicken genome (Gallus_gallus-5.0) through HISAT2 2.1.0 [28] (Additional file 1 Table S1).

### Genomic characterization and classification of lncRNAs

**Fig. 1:**
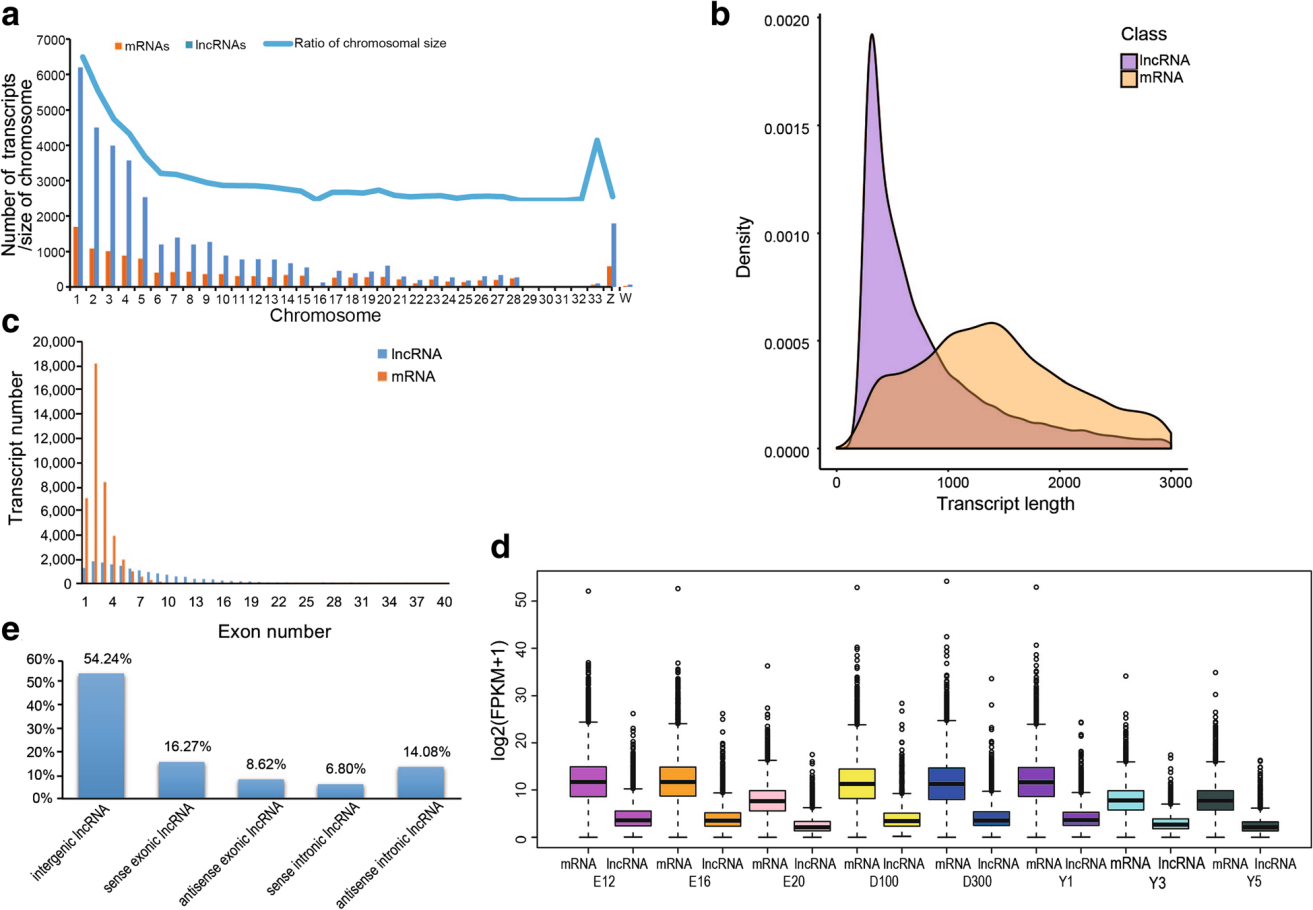
Genomic characterization of lncRNAs. **a** Chromosome distribution of mRNA and lncRNA transcripts identified in chicken cerebrum. The blue line represents the size of chromosome (the ratio of each chromosomal size to the total genomic size). **b** The distribution of transcript length of lncRNAs and mRNAs. **c** Exon number distribution of lncRNAs and mRNAs. **d** Comparison of the expression levels of lncRNAs and mRNAs. The lines of the whiskers in the box represent median lines. **e** The classification of lncRNAs

To compare the genomic characteristics of lncRNAs and mRNAs, we identified a total of 39,907 putative lncRNAs and 14,558 mRNAs that were expressed in more than one biological replicate (FPKM > 0). Subsequently, the genome distribution of lncRNAs and mRNAs was investigated, of which there were 36,427 (86.04%) transcripts and 12,254 (93.02%) mRNAs were assembled on chromosomes (Fig. 1a and Additional file 1 Table S2). Most of the lncRNAs and mRNAs of chicken tended to distribute in large chromosomes (No. 1–10 and Z chromosome), while a small proportion of transcripts distributed in micro chromosomes. In addition, the number of lncRNAs and mRNAs were highly correlated with chromosome size (*r* = 0.9850, 0.9677, respectively). Illustrating that the number of transcripts distributed on chromosomes was proportional to the size of chromosome. Based on further comparisons, we found that chicken lncRNAs are approximately 1/2 the length of mRNAs (mean length of 1122 nt for lncRNAs and 2029 nt for mRNAs); moreover, lncRNAs have fewer exons and are expressed at lower levels than mRNAs (Fig. 1b-d).

We identified 36,663 lncRNAs and classified them by genomic location; 54.24% of them were classified as intergenic lncRNAs not overlapping with any genes, 16.27% as sense exon overlapping lncRNAs, 8.62% as antisense exon overlapping lncRNAs, 6.80% as sense intron overlapping lncRNAs and 14.08% as antisense intron overlapping lncRNAs (Fig. 1e and Additional file 1 Figure S1). Similarly, the subgroups of lncRNAs (intergenic, exonic and intronic lncRNA) distributed on chromosomes in proportion to the chromosome size (Additional file 1 Table S2 and Figure S2). Furthermore, we performed functional enrichment analysis on both exon overlapping lncRNAs and intron overlapping lncRNAs, terms related to neuron, axon and synapse were found to be enriched in these genes (Additional file 2). For instance, GO: 0043524 (negative regulation of neuron apoptotic process), GO: 0048666 (neuron development) and GO: 0007269 (neurotransmitter secretion). Axon activities including GO: 0007411 (axon guidance), GO: 0008089 (anterograde axonal transport), GO: 0031290 (retinal ganglion cell axon guidance), GO: 0019896 (axonal transport of mitochondrion), GO: 1904115 (axon cytoplasm) and GO: 0071679 (commissural neuron axon guidance). Terms involved in synaptic regulation contained GO: 0045211 (postsynaptic membrane), GO: 0051965 (positive regulation of synapse assembly), GO: 0007416 (synapse assembly), GO: 0048813 (dendrite morphogenesis). KEGG pathways were primarily involved in cell proliferation and differentiation, development such as “Insulin signaling pathway”, “MAPK signaling pathway”, “ErbB signaling pathway”, “Hedgehog signaling pathway”; “Neuroactive ligand-receptor interaction” and “Calcium signaling pathway” were related to neuron. These results indicated that the intron and exon overlapping lncRNA functions were consistent with the biological functional activities of the genes enriched in the cerebrum.

### Expression profiles of mRNAs and lncRNAs

**Fig. 2:**
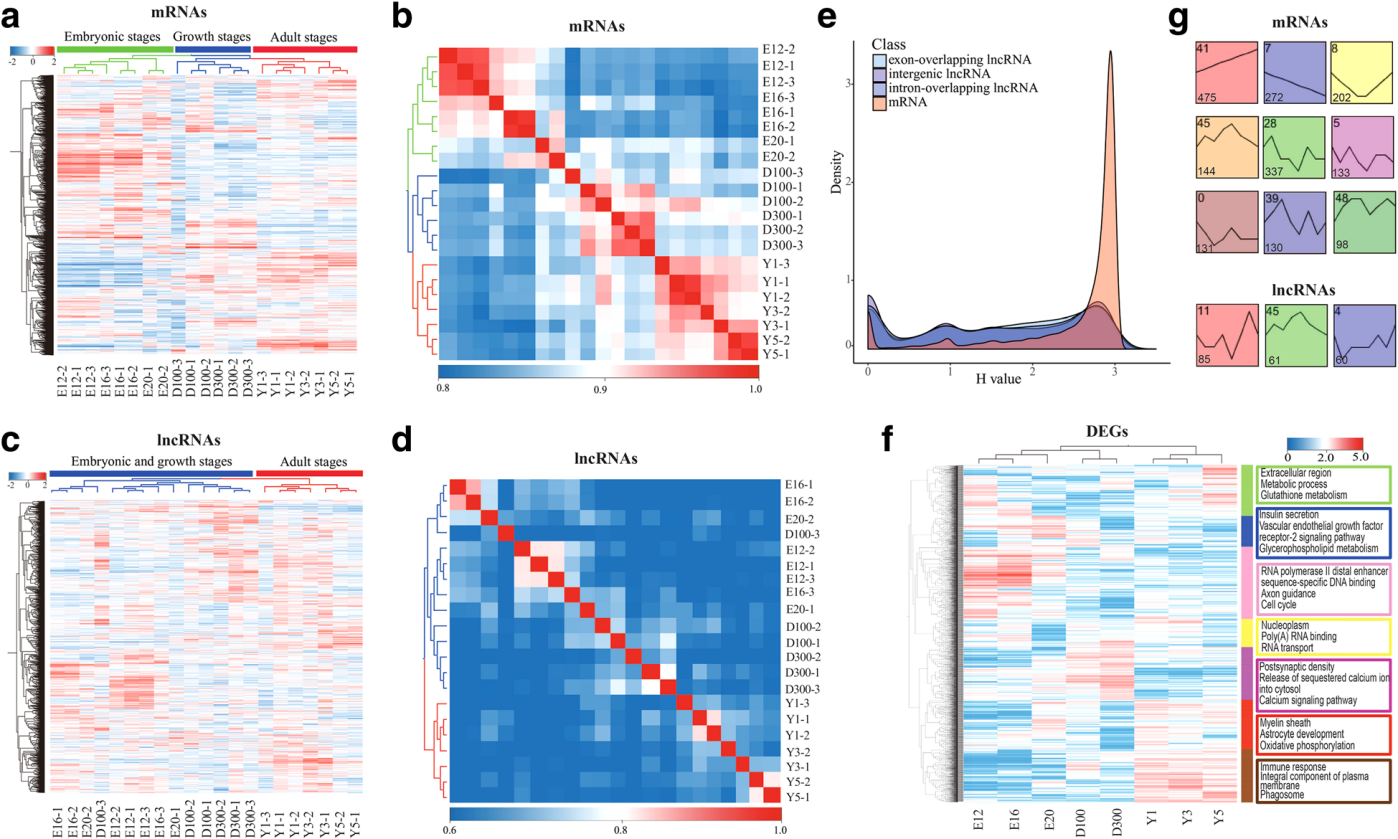
The expression profiles of mRNAs and lncRNAs. **a**, **c** The expression profiles of mRNAs (upper panel) and lncRNAs (lower panel). The heat map shows the expression profile of mRNAs and lncRNAs. The top and left panel is the sample and gene tree constructed by Pearson Correlation; the value represents the log2 transformed values of (FPKM+ 1). **b**, **d** Dynamic changes in expression profile of mRNAs and lncRNAs. The value represents the pairwise Pearson Correlation. The correlation between every two samples was calculated by log2-transformed (FPKM+ 1) gene expression values. **e** The distributions of Shannon entropy-based temporal specificity scores that were calculated for distinct classes of mRNAs and lncRNAs. **f** Age-dependent clusters with significantly enriched GO terms and pathways map. Hierarchical clustering analysis grouped the age-dependent genes into 7 clusters. **g** Temporal profiles of age-dependent DEGs (upper panel) and DE lncRNAs (lower panel). Modules in different colors represent different temporal expression patterns which were significantly enriched by STEM analysis (non-significant modules were not shown). Numbers in the top indicates the module number and the lower numbers indicate numbers of DEGs and DE lncRNAs in each module

Our RNA-Seq experiments included a broad time series of sampling which allowed us to follow the expression dynamics of mRNAs and lncRNAs as development proceeded. Based on the expression dynamics of mRNAs and lncRNAs, the Pearson correlation between each pair of samples was calculated. The results showed that 21 samples were grouped into three broad clusters of mRNA expression profiles (Fig. 2a): the first cluster (green) represented the embryonic stages (E12, E16 and E20), the second cluster (blue) meant the growth stages (D100 and D300) and the last cluster (red) was the adult stages (Y1, Y3 and Y5). Chickens included in the red cluster had reached a growth plateau and even begun the ageing process. While the expression profile of lncRNAs was not as markedly varied as that of mRNAs, the embryonic and growth stages were clustered, and the adult stages were congregated (Fig. 2c). In addition, the correlation power of mRNAs at consecutive time points exhibited strong time-dependent clustering. Meanwhile, the correlation power of lncRNAs between stages of lncRNAs was much weaker (Fig. 2b, d). Subsequently, we calculated the Shannon entropy (H) value [29] to further test the hypothesis; this parameter can be used to measure the specificity of gene expression during developmental stages. Compared with mRNAs, namely, lincRNAs, intron overlapping and exon overlapping lncRNAs, exhibited increased temporal specificity (Fig. 2e).

### Age-dependent transcriptome and gene expression analysis

To evaluate differentially expressed lncRNAs and mRNAs (DE lncRNAs and DEGs) at irregularly spaced sampling times, we compared the expression levels at adjacent time points with the threshold of FC(Fold Change) ≥ 2 (or ≤ 0.5) plus a Bonferroni-adjusted *P*-value ≤ 0.05. Overall, we compared 7 pairs and identified 1892 unique lncRNAs and 4499 unique mRNAs as significantly differentially expressed during development and ageing. In the lncRNAs comparison libraries, 1122 and 1059 lncRNAs were found to be up- and down-regulated, respectively; a total of 2985 up-regulated and 2993 down-regulated DEGs were identified in mRNAs comparison groups during chicken cerebrum development and ageing. The greatest number of DE lncRNAs between adjacent time points was observed at Y5 vs Y3, followed by Y1 vs D300 and E16 vs E12, while the most DEGs were detected at Y1 vs D300, followed by E20 vs E16 and E16 vs E12 (Additional file 1 Figure S3).

To further investigate the biological functional variations of these DEGs in development and ageing, we used 4499 DEGs to perform hierarchical clustering analysis. The sample tree was clustered into 3 subgroups by age, and the gene tree was partitioned into 7 clusters, which were labeled with different colors (Fig. 2f). Subsequently, we performed functional enrichment analysis based on the DEGs of the 7 clusters. Generally, DEGs expressed highly in embryonic periods were enriched in the terms “cell division”, “insulin secretion” and “axon guidance”; the most relevant KEGG pathways were “glutathione metabolism”, “Notch signaling pathway” and “cell cycle” (the first three clusters). DEGs in growth periods were enriched in the terms “MAPK signaling pathway”, “GnRH signaling pathway” and “Calcium signaling pathway” (the two middle clusters). Adult periods were mainly enriched in immune response, phagosome and defense response to virus (the last 2 clusters) (Additional file 3).

### Temporal profiles of gene expression

We used STEM to group DEGs with similar temporal profiles and found that 4340 mRNAs and 1218 lncRNAs were classified into time-series expressed patterns. For mRNAs, a total of 1922 (44.29%) genes were partitioned into 9 significantly enriched temporal profiles (*P* ≤ 0.05). Two models exhibited monotonic trajectories: the pink and blue-violet models showed a gradual increase and decrease with age, respectively. Meanwhile, a portion of genes were assigned to models that reversed their trajectory over time: the yellow model presented a U-shaped trajectory with a minimum at D100 to D300 and the orange model showed a Bell-shaped trajectory with a peak at D300. The remaining models had more complex temporal profiles, some with an initial increase (green, light purple and jade models) and some with an initial decrease in their expression (mauve and light brown models) (Fig. 2g). As for lncRNAs, 206 (16.91%) transcripts were assigned to significantly enriched model profiles (*P* ≤ 0.05) (Fig. 2g). To investigate the biological functions of these significantly enriched mRNAs, we performed functional enrichment analysis on aforementioned genes in 9 models. Interestingly, the terms “cytokine receptor activity” (GO: 0004896), “phagosome” and “ECM-receptor interaction” were enriched in increasing genes; “cell cycle”, “MAPK signaling pathway”, “p53 signaling pathway”, “mitotic nuclear division” (GO: 0007067), “axon guidance” (GO: 0007411), “axon hillock” (GO: 0043203) and “cell proliferation” (GO: 0008283) were enriched in decreasing genes. In addition, “activation of GTPase activity” (GO: 0090630) and “regulation of axonogenesis” (GO: 0060070) were enriched in DEGs from Bell-shaped temporal profiles; “canonical Wnt signaling pathway” (GO: 0060070) and “growth” (GO: 0040007) were enriched in DEGs from U-shaped temporal profiles (Additional file 4).

### Construction of a co-expression network

Predicting the putative function of lncRNAs through sequence characteristics is still a challenge because of the lack of annotated features. WGCNA can be applied to associate lncRNAs with functionally annotated mRNAs because genes and transcripts with similar expression patterns are often related in biological functions and can be attributed to the same module. We first used DESeq2 to screen out mRNAs and lncRNAs; secondly, we conducted WGCNA to explore the function of lncRNAs, a total of 11,200 mRNAs and 955 lncRNAs were selected by screening to construct the co-expression network. Ultimately, 12 modules were identified; the top 2 modules accounted for 74.02 and 70.26% of the total mRNAs and lncRNAs, respectively. We therefore considered a total of 8323 mRNAs were targeting genes to 671 lncRNAs. Furthermore, the co-expressed genes of the top 2 modules (8323 mRNAs) were submitted to DAVID; the functional categories showed that these co-expressed genes were significantly enriched in the terms “myelin sheath” (GO: 0043209), “dendrite morphogenesis” (GO: 0048813) and “axonogenesis” (GO: 0007409); as well as nuclear components such as “nucleolus” (GO: 0005730), “nucleoplasm” (GO: 0005654), “chromatin organization” (GO: 0006325). Consistently, KEGG analysis showed an extensive enrichment of co-expressed genes involved in pathways such as “spliceosome”, “ribosome”, “cell cycle”, “insulin signaling pathway” and “mTOR signaling pathway”. It is worth noting that, pathways related to defense, detoxication and antiageing were significantly enriched in adult stages: “positive regulation of telomere maintenance via telomerase” (GO: 0032212). “Toxin transport” (GO: 1901998), “damaged DNA binding” (GO: 0003684), “wound healing” (GO: 0042060), “glutathione metabolic process” (GO: 0006749) and “response to UV” (GO: 0009411) (Additional file 5).

### Validation of the gene expression by qPCR

**Fig. 3:**
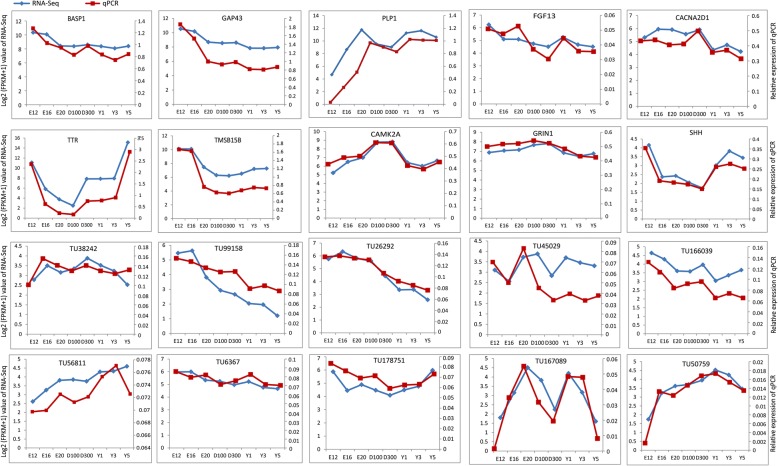
Validation of 10 expressed DEGs and DE lncRNAs by qPCR. The X-axis represented 8 developmental stages. The Y-axis indicated the relative expression of each gene; blue lines were log_2_ (FPKM+ 1) values of RNA-Seq and the red lines were relative expression of qPCR

To validate the sequencing data, we selected 10 mRNAs and lncRNAs representing different temporal profiles to examine the expression patterns at each developmental stage. Three DEGs (*TMSB15B*, *BASP1*, *GAP43*) were decreasing pattern, 2 DEGs were increasing (*FGF13*, *CACNA2D1*), Bell-shaped (*CAMK2A*, *GRIN1*) and U-shaped (*TTR*, *TMSB15B*) patterns, respectively, and 1 DEG (*PLP1*) came from the initial-up expressing pattern. Correspondingly, 3 DE lncRNAs in pink module (TU56811, TU178751, TU167089), 3 in green module (TU99158, TU26292, TU50759), and 4 in blue module (TU38242, TU45029, TU166039, TU6367) were selected from the temporal expression profiles of lncRNAs in Fig. 2g. The relative expression of 10 mRNAs and lncRNAs detected by qPCR were compared with the transformed log_2_ (FPKM+ 1) values of RNA-Seq. The results showed that the altered expression patterns of 10 mRNAs and lncRNAs at each stage were consistent with the RNA-Seq data (Fig. 3), illustrating the reliability of our RNA-Seq data and guaranteed the accuracy of stringent pipeline was used to identify the transcripts.

## Discussion

### Genomic characterization of lncRNAs in chicken cerebrum

Although the brain lncRNA profile of human, macaque, mice and fish have been well characterized in recent years, the function of lncRNAs in chicken brain development and ageing remained unclear. Here, we systematically investigated the lncRNA profile of the cerebrum in female Tibetan chicken by RNA-Seq spanning 8 stages from embryonic (E12) to ageing (Y5) individuals. We obtained 406.56 Gb of raw data from 21 libraries in total; the chromosomal distribution of transcripts illustrated that the number of transcripts distributed on chromosomes was proportional to the size of chromosome; not surprisingly, several key genomic characteristics of the chicken cerebrum that lncRNAs were shorter, less conserved and expressed at lower levels than mRNAs were consistent with results in zebrafish and mammals [2, 8, 9, 17, 18]. Furthermore, we identified approximate 2000 (54.24%) lincRNAs (long intergenic lncRNAs) in our study, accounting for more than half of the total chicken lncRNAs, which were consistent with previous studies [30, 31]. Functional enrichment analysis indicated that the functions of intron and exon overlapping lncRNAs were consistent with the biological functional activity of the genes that were enriched in the cerebrum, and that brain transcriptomes are conserved across vertebrates.

### Atlas of gene expression in chicken cerebrum

The expression profile of mRNAs of the chicken cerebrum was age-dependent and lncRNA has a more restricted temporal expression than mRNA, suggesting that there might be distinctive patterns of age-related functional changes across cerebrum developmental stages. Further findings on age-dependent transcripts showed that DEGs were grouped into 7 sub clusters. The first 3 clusters mainly highly expressed in embryonic stages, involving in the formation of neuronal network and development. For example, *HDAC2* encodes histone deacetylase which can form transcriptional repressor complexes by associating with many different proteins, and play an important role in transcriptional regulation, cell cycle progression and developmental events. Previous reports have indicated that *HDAC2* is critical for oocyte development and reciprocally, it also can maintain normal chromatin structures and has a unique role by controlling the fate of neural progenitors during normal brain development [32, 33]. Activity of protein product encoded by *PAX6* is essential in the development of neural tissues, particularly the eye. *Pax6* can establish distinct ventral progenitor cell populations and control the identity of motor neurons and ventral interneurons [34, 35]. The 2 middle clusters were up-regulated at growth stages. It was noted that, GnRH signaling pathway were enriched in this stage alone, the GnRH secreted from the hypothalamus acts upon its receptor in the anterior pituitary to regulate the production and release of the gonadotropins, LH and FSH [36, 37], which stimulated the hormones secretion and egg formation in ovary. This result was in accordance with the egg laying traits in Tibetan chicken that they began to lay eggs at the age of 240 to 270 days and reached egg laying peak at the age of 300 days [38]. Besides, GnRH could activate the signaling of PCK (protein kinase C) and lead to transactivation of the epidermal growth factor (EGF) receptor and activation of mitogen-activated protein kinases (MAPKs), resulting in activation of transcription factors and rapid induction of early genes [37]. This coincided with the fact that MAPK signaling pathway was significantly enriched in growth stage. Mainly up-regulating genes in the last 2 clusters of adult stages exerted functions in defense mechanisms and immune responses. *CX3CL1* is broadly expressed in brain, it plays a role in a wide range of diseases, including cancer, vasculitis, neuropathies, atherosclerosis, inflammatory diseases, and in human immunodeficiency virus infections [39, 40]. The protein encoded by *MARCO* is part of the innate antimicrobial immune system [41], and modulates inflammatory responses against virus infection [42]. All these findings elucidated that biological functions of highly expressed genes in different clusters accorded with the development and growth rules.

### Monotonic temporal expression patterns

The progressively increasing gene expression profile involved in phagosome and binding, which meant that organisms were undergoing neurodegeneration, anti-inflammation and innate immune mechanisms were activated as the neurons aged [12, 43, 44]. Meanwhile, the temporal profile decreased with time, of particular interest, were the down-regulation of cell proliferation and mitotic nuclear division and the degeneration of neuronal projections and axonal activity. In addition, chromatin and extracellular matrix remodeling occurred in this gene cluster. Similarly, neuronal functional deterioration and synaptic down-regulation were differentially represented in the ageing primate brain [45]. All the information indicated that a progressive accumulation of immune function and neuronal deterioration were conserved hallmarks of brain ageing.

### Temporal inversions

Interestingly, a substantial proportion of DEGs reversed their expression trends at D300 and exhibited U-shaped or Bell-shaped temporal profiles. The U-shaped model applied to the “canonical Wnt signaling pathway” and “glutathione metabolism”; Wnt signaling is involved in organogenesis, and the inhibition of Wnt signaling is an essential factor in the late stage of body profile formation in vertebrates [46]. Glutathione is helpful in maintaining a normal immune system and has an antioxidant effect [2], hinting that the Wnt signaling pathway might be mapped to genes down-regulated from E12 to D100, while glutathione metabolism probably corresponds to genes up-regulated from D300 to Y5. The Bell-shaped expression model exhibited a peak at D300 and a sub-peak at E16; this gene cluster was enriched for “GTPase activity”, “regulation of axonogenesis” and “neuronal synaptic plasticity”. GTPase participated in protein biosynthesis and translocation, cell cycle control and cell differentiation [47]. Axonogenesis is a subfield of neural development concerning the process by which neurons send out axons to reach the correct targets [48]. In neuroscience, synaptic plasticity is the ability of synapses to strengthen or weaken over time, in response to increases or decreases in their activity; this phenomenon is one of the important neurochemical foundations of learning and memory [49]. Our results suggest that genes in this model were initially involved in development, while the oldest group exhibited a reduction of brain function; in other words, chicken cerebrum senescence was accompanied by impairment of neural plasticity, increases in inflammatory response and neurological disorders, and decrease of learning and memory abilities. Generally, hens at the age of 300 days are high egg productive, subsequently, their physiological features change substantially with age, leading to a reproductive decline and a drop in egg laying. Therefore, we can detect the biological variations in RNA levels.

### Different complex temporal expression patterns

Genes in the green model entered periods of up-regulation during embryonic development (E12 to E16) and adulthood (D300 to Y1). On embryonic days 12 to 16, when histological differentiation is gradually completed, these up-regulated genes were enriched for the terms “myelin sheath” and “synaptic vesicle endocytosis”, indicating neural functions were important in this stage. Even so, the immune response was fully activated; for instance, the terms “lysosome”, “phagosome”, “complement activation”, “response to oxidative stress” and “T cell receptor signaling pathway” were enriched. It is well known that 300-day-old hens are adults, but we additionally detected signs of ageing at D300 to Y1, implying that the gene expression changes preceded physical ageing.

### Functional prediction of lncRNAs

It is well known that lncRNAs are important regulatory factors exerted their roles by targeting corresponding mRNAs [50]. To gain insight into how putative lncRNAs performed the biological functions to regulate the cerebrum development and ageing, co-expression networks between DE lncRNAs and DEGs were constructed. Functional enrichment analysis of co-expressed genes showed similar results, except for pathways involved in the formation of neuron network, cell division and development, genes such as *SYT1*, *CDH1*, *PAX6*, *NEFL*, and *ATXN*, which are typical neuronal markers in brain development [2] were discovered in this study. Besides, GO terms and KEGG pathways related to defense mechanisms and immune responses were enriched, implying the biological function of DE lncRNAs in chicken cerebrum in regulating the brain developmental and immunological mechanisms.

## Conclusions

In summary, we have identified a set of age-dependent lncRNAs and mRNAs, determined their temporal expression patterns and clarified the dynamic changes in the chicken cerebrum transcriptome over the course of time. Although some important genes and pathways were reported in this study, no work has been conducted on the functions of lncRNAs in chicken cerebrum, the molecular genetic mechanisms regulating chicken brain development and ageing are still poorly known. Therefore, to validate studies based on these hypothetical results deduced by bioinformatics methods were needed. Collectively, these findings may contribute to further studies on the molecular mechanisms of chicken development. In addition, as the chicken is an important model organism bridging the evolutionary gap between mammals and other vertebrates [51], our results may help to improve our comparative understanding of the cerebrum during vertebrate evolution.

## Materials and methods

### Ethics statement

The experimental procedures in this study were approved by the Committee on the Care and Use of Laboratory Animals of the State-level Animal Experimental Teaching Demonstration Center of Sichuan Agricultural University (Approval ID: B20160403). Anesthetization was performed in accordance with the Regulations for the Administration of Affairs Concerning Experimental Animals (China, revised 2004), phenobarbital sodium solution with the concentration of 30 mg/kg was intraperitoneally injected and all efforts were made to minimize the suffering of the chickens. Finally, chickens were euthanized with overdose of phenobarbital sodium.

### Chick embryo incubation and sample collection

Fertile eggs (Tibetan chicken) were bought from Mao County Jiuding Original Ecological Livestock and Poultry Breeding Co., Ltd., Aba Autonomous Prefecture, Sichuan Province. Fertile eggs were incubated at 37.8 °C (incubation days of 1–19) and 37.2 °C (incubation days of 20–21) with 70–80% humidity; a 24 h lighting schedule and intermittent rotations were employed. Ten chick embryos were randomly obtained on embryonic days 12, 16, and 20 (E12, E16 and E20). We sampled cerebrums for further high-throughput sequencing and skeletal muscle for sex determination. All chicks were housed in the same room from hatching to 6 weeks of age at a density of 12 chicks /m^2^ under uniform environmental conditions at Ya’an Poultry Farm, which belongs to Sichuan Agricultural University. After that, chicks were housed in cages, the uniformed poultry integration cage systems were established with 2 storeys, the size of each unit was set as 2.5 m length × 1.8 m width × 0.8 m height, and the density of stock was controlled to be 10 chicks /m^2^. All food, water, temperature, and light condition were monitored digitally. Healthy female hens were sacrificed at the age of 100 and 300 days (D100 and D300), together with hens aged 1, 3 and 5 years, cerebrum tissues were collected from 3 biological replicates at each time point except for E20, Y3 and Y5 (2 replicates). It was noted that, we collected 21 samples across 8 developmental stages in total, only 2 female embryos were identified among samples collected at the embryonic day 20, in order to collect samples in aging (Y5) as many as possible, the left hens were divided into 2 groups at Y3 and Y5 with 2 replicates, respectively. All the samples were snap frozen in liquid nitrogen immediately after collection and then stored at − 80 °C until RNA extraction.

### Embryonic sex determination by PCR

The embryos were sexed by PCR, which was performed to amplify the CDH1-Z/W sequence. We designed *CHD1*-based primers because some introns of *CHD1* differ between the Z and W chromosomes [52]. The sequence of the forward primer was 5′-GTTACTGATTCGTCTACGAGA-3′ and that of the reverse primer was 5′-ATTGAAATGATCCAGTGCTTG-3′. The PCR program was as follows: 5 min at 94 °C; 35 s at 94 °C, 30 s at 54 °C, and 30 s at 72 °C for 40 cycles; 5 min at 72 °C for final extension. Electrophoresis was performed on 1.5% agarose gel to identify the PCR products; males were identified by the presence of one band (*CHD1-Z* band), while females were identified by the presence of 2 bands (*CHD1-Z* and *CHD1-W* bands); female chicks were selected from every stage to use for high-throughput sequencing.

### RNA isolation, library construction and Illumina deep sequencing

We used TRIzol Reagent (Invitrogen, Carlsbad, CA, USA) to isolate total RNA following the manufacturer’s instructions; genomic DNA was removed by DNaseI (Qiagen, Beijing, China). The RNA purity was determined by measuring absorbance at 260 nm and 280 nm on an ND-1000 spectrophotometer (NanoDrop 2000, Thermo Fisher Scientific, Waltham, MA, USA) to calculate the A260/280 ratio; the integrity and concentration were estimated using Agilent 2100 Bioanalyzer (Agilent Technologies, Palo Alto, Calif.) with the Agilent RNA Nano Kit. In addition, RNA integrity was examined by 1% agarose gel electrophoresis. Samples with RIN (RNA integrity number) values above 7.5 and 28S/18S ≥ 1.0 were used for library construction and sequencing. The Ribo-Zero™ Gold Kit (Illumina, San Diego, CA, USA) was used to remove rRNA from the total RNA. Subsequently, the sequencing libraries were generated following manufacturer recommendations with varied index label by NEBNext® Ultra™ Directional RNA Library Prep Kit for Illumina (NEB, Ispawich, USA). The details of library construction showed as follow: Firstly, rRNA was removed by kits, RNA fragmentation and short RNA strands were carried out by NEBNext First Strand Synthesis Reaction Buffer under elevated temperature. Subsequently,first cDNA strand was synthesized using random hexamer primers and RNA fragments as template. Second strand cDNA synthesis was subsequently performed using buffer, dNTPs, DNA polymerase I and RNase H. The library fragments were purified with QiaQuick PCR kits and elution with EB buffer, then terminal repair, poly (A)-tailing and adapter ligation were implemented. In order to select cDNA fragments of preferentially 300 bp in length, the library fragments were purified and the UNG enzyme was used to digest second strand of cDNA. PCR was performed by aiming expected size of amplicons, and the library was completed. The resulting 21 libraries were sequenced using the Illumina HiSeq platform with a paired-end sequencing length of 150 bp (PE150) at Annoroad Gene Technology Corporation (Beijing, PR China).

The clean data have been submitted to the NCBI Gene Expression Omnibus (GEO) with the accession number GSE114129.

### Read mapping, quantification and transcriptome annotation

The quality control of raw data was completed by removing low-quality reads, adaptor sequences, empty reads and rRNA reads. Processed reads from each sample were mapped to the chicken reference genome (Gallus_gallus-5.0) using HISAT2 2.1.0 followed the previous described protocol [28, 53]. The mapped reads were assembled with StringTie v1.3.3 [54]; assembled transcripts were then merged into consensus transcripts using custom Python scripts [55] after filtering reads with length less than 200 nt. Finally, transcripts annotated as “c” and “=” were removed by Cuffcompare v2.2.1, noted that, “c” for partial match, “=” for full match [56]. Then, we obtained a set of putative coding and non-coding transcripts, and the known transcripts were removed by blastx and Hmmscan [57].

### Identification and classification of lncRNAs

The Coding Potential Calculator (CPC) was applied to estimate the coding ability of the remaining transcripts, and transcripts with CPC score > 0 were filtered [58]. Subsequently, transcripts with FPKM > 0 at least in one biological replicate were identified as lncRNAs (Noted that FPKM is abbreviation of fragments per kilobase of transcript per million mapped reads). LncRNAs were initially classified into 2 major categories by FEELnc [59] according to their location with respect to mRNA: (1) intergenic lncRNAs, which do not intersect with any gene annotations; (2) intragenic lncRNAs, which overlap with gene annotations and can be further classified into 4 subcategories: (a) lncRNAs that overlap with sense introns, (b) lncRNAs that overlap with antisense introns, (c) lncRNAs that overlap with sense exons, and (d) lncRNAs that overlap with antisense exons. The intragenic lncRNAs occurred in two situations: either the lncRNA contained the intron or exon, or the lncRNA was contained in the intron or exon.

### Transcriptomic gene expression analysis

The expression levels of the transcripts were expressed as FPKM values of mRNA and lncRNA using Cufflinks v2.2.1 [56] and StringTie v1.3.3 [54], where a value of 1 was added to the FPKM value of each gene before log_2_ transformation to avoid infinite values. FPKM > 0.1 was used to filter the expressed mRNA [60]. Pearson correlations were estimated across 8 developmental stages, and hierarchical clustering was performed using MultiExperimentViewer (MeV version 4.9.0) [61].

### Age-dependent transcriptome and temporal expression pattern analysis

To test age-dependent differential gene expression at 8 time points, we identified differentially expressed mRNAs and lncRNAs using DESeq2 [62] based on the read counts produced from the FPKM calculated by StringTie v1.3.3 [54]. We conducted pairwise comparisons between adjacent time points. Genes and transcripts with FC ≥ 2 or ≤ 0.5 plus *P*-value ≤ 0.05 were identified as age-dependent genes. For instance, in the E16 vs. E12 comparison (the latter time point was defined as the numerator), a gene with the FC ≥ 2 would be grouped into the increased pattern and considered to be up-regulated during this stage, a gene with FC ≤ 0.5 would be grouped into the “decreased pattern”, and considered to be down-regulated. To evaluate the time course of age-dependent transcriptomic variations across the life cycle of the chicken, we performed STEM analysis (Short Time-series Expression Miner) based on differentially expressed genes and transcripts by comparing 2 adjacent time points [63]. Different colors were used to represent the significantly enriched model profiles (Bonferroni-adjusted *P*-values ≤ 0.05).

### LncRNA-mRNA co-expression network analysis

We constructed a co-expression network using WGCNA (Weighted Gene Co-expression Network Analysis) in the R package [64] based on the differentially expressed mRNAs and lncRNAs that were identified by DESeq2 screening. Genes with coordinated expression patterns were termed “co-expression modules” and were detected by the dynamic tree cut method [65]. Functional annotation enrichment analyses of Gene Ontology (GO) and Kyoto Encyclopedia of Genes and Genomes (KEGG) terms were conducted with the DAVID (Database for Annotation, Visualization, and Integrated Discovery) dataset (https://david.ncifcrf.gov) [66]. GO terms and KEGG pathways with *P*-values ≤ 0.05 were considered significantly enriched.

### Validation of the gene expression by qPCR

We selected 10 DEGs and DE lncRNAs to validate the expression profiles of RNA-Seq by relative quantification/Real-Time PCR (q-PCR). The expression levels of the selected genes were normalized to β-actin, primers for the mRNAs/lncRNAs were designed using Primer-BLAST (https://www.ncbi.nlm.nih.gov/tools/primer-blast/) (Additional file 1 Table S3). Total RNA was converted to cDNA using the EasyScript One-Step gDNA Removal and cDNA Synthesis SuperMix (Transgen Biotech, China). The qPCR was performed on the Bio-Rad iQ5 Real-Time PCR Detection system to detect the RNA expression using TransStart Top Green qPCR SuperMix (Transgen Biotech, China). The reaction volume contained 5 μl TransStart Top Green qPCR SuperMix, 1 μl template cDNA, 0.4 μl forward and reverse primers, and added ddH_2_O to the final volume of 10 μl. Each sample was conducted with three technique replicates followed the qPCR system: 94 °C for 30 s, followed by 40 cycles of 94 °C for 5 s and 30s at the Tm indicated in Additional file 1 Table S3. The melting curve analysis was performed from 65 °C to 95 °C with increments of 0.5 °C for 5 s. The relative expression levels were calculated using the 2^-△△Ct^ method, and the data were expressed as mean ± SD (standard deviation).

## Additional files

Additional file 1: **Table S1.** Data statistics of samples for RNA sequencing. **Table S2.** Number of mRNA and lncRNA (including exonic, intronic and intergenic lncRNA) transcripts distributed on chromosome identified in chicken cerebrum. **Table S3.** Primer sequences for qPCR. **Figure S1.** Pipeline of lncRNAs identification. **Figure S2.** Chromosome distribution of 3 subgroups of lncRNAs. The blue line represents the size of chromosome (the ratio of each chromosomal size to the total genomic size). The Pearson correlation of chromosomal size and exonic lncRNA, intronic lncRNA and intergenic lncRNA were 0.0960, 0.9789 and 0.9874, respectively. **Figure S3.** Age-dependent mRNAs (A) and lncRNAs (B). Age-dependent genes in each stage were identified through comparing 2 adjacent time points, genes and transcripts with the FC ≥ 2 or ≤ 0.5 plus the *P*-value ≤ 0.05 were identified as age-dependent genes. (DOC 295 kb)

Additional file 2: Functional categories of genes overlapping with intronic and exonic lncRNAs. (XLSX 27 kb)

Additional file 3: Top 10 significantly enriched categories of age-dependent genes in 7 clusters. (XLSX 21 kb)

Additional file 4: Functional categories of genes based on significantly enriched models in STEM analysis. (XLSX 23 kb)

Additional file 5: Functional categories of co-expressed genes in the top 2 modules. (XLSX 41 kb)

## Abbreviations

DE lncRNAs: Differentially expressed lncRNAs
DEGs: Differentially expressed genes
FPKM: Fragments per kilobase of transcript per million mapped reads
GEO: Gene Expression Omnibus
GO: Gene Ontology
KEGG: Kyoto Encyclopedia of Genes and Genomes
STEM: Short Time-series Expression Miner
WGCNA: Weighted Gene Co-expression Network Analysis
