# Supplementary material for: The temporal expression patterns of brain transcriptome during chicken development and ageing
Source: BMC Genomics. 2018 Dec 13;19:917. doi: 10.1186/s12864-018-5301-x (PMC6293534; doi:10.1186/s12864-018-5301-x)
Supplement: Supplementary file 1 — Table S1. Data statistics of samples for RNA sequencing. Table S2. Number of mRNA and lncRNA (including exonic, intronic and intergenic lncRNA) transcripts distributed on chromosome identified in chicken cerebrum. Table S3. Primer sequences for qPCR. Figure S1. Pipeline of lncRNAs identification. Figure S2. Chromosome distribution of 3 subgroups of lncRNAs. The blue line represents the size of chromosome (the ratio of each chromosomal size to the total genomic size). The Pearson correlation of chromosomal size and exonic lncRNA, intronic lncRNA and intergenic lncRNA were 0.0960, 0.9789 and 0.9874, respectively. Figure S3. Age-dependent mRNAs (A) and lncRNAs (B). Age-dependent genes in each stage were identified through comparing 2 adjacent time points, genes and transcripts with the FC ≥ 2 or ≤ 0.5 plus the P-value ≤ 0.05 were identified as age-dependent genes. (DOC 295 kb) [file 12864_2018_5301_MOESM1_ESM.doc]

**Additional file 1: additional tables and figures**

**Tables**

**Table S1.** Data statistics of samples for RNA sequencing

| **Sample description (age)** | **Sex** | **Tissue** | **Sequencing strategy** | **Sample ID** | **Raw reads number** | **Clean reads number** | **Clean reads rate (%)** | **Raw data (Gb)** | **Clean data (Gb)** | **Clean reads rate (%)** | **Clean Q30 bases rate (%)** | **Overall alignment rate (%)** |
| --- | --- | --- | --- | --- | --- | --- | --- | --- | --- | --- | --- | --- |
| Embryonic day 12 | Female | Cerebrum | PE150 | E12-1 | 129,684,834 | 127,027,030 | 97.95 | 19.45 | 19.05 | 97.95 | 92.59 | 94.24 |
|  |  |  |  | E12-2 | 151,493,072 | 148,260,550 | 97.87 | 22.72 | 22.24 | 97.87 | 92.18 | 94.61 |
|  |  |  |  | E12-3 | 162,967,018 | 158,989,938 | 97.56 | 24.45 | 23.85 | 97.56 | 91.97 | 94.07 |
| Embryonic day 16 | Female | Cerebrum | PE150 | E16-1 | 148,652,000 | 145,676,244 | 98.00 | 22.30 | 21.85 | 98.00 | 93.75 | 93.78 |
|  |  |  |  | E16-2 | 121,871,672 | 119,801,724 | 98.30 | 18.28 | 17.97 | 98.30 | 93.60 | 94.33 |
|  |  |  |  | E16-3 | 139,343,200 | 137,026,924 | 98.34 | 20.90 | 20.55 | 98.34 | 93.84 | 94.23 |
| Embryonic day 20 | Female | Cerebrum | PE150 | E20-1 | 140,775,956 | 138,271,340 | 98.22 | 21.12 | 20.74 | 98.22 | 92.53 | 94.25 |
|  |  |  |  | E20-2 | 120,367,592 | 118,529,776 | 98.47 | 18.06 | 17.78 | 98.47 | 92.39 | 94.33 |
| 100 days old | Female | Cerebrum | PE150 | D100-1 | 109,506,844 | 107,605,434 | 98.26 | 16.43 | 16.14 | 98.26 | 92.07 | 93.27 |
|  |  |  |  | D100-2 | 120,549,454 | 118,315,670 | 98.15 | 18.08 | 17.75 | 98.15 | 92.13 | 93.82 |
|  |  |  |  | D100-3 | 131,803,556 | 129,635,162 | 98.35 | 19.77 | 19.45 | 98.35 | 95.16 | 94.24 |
| 300 days old | Female | Cerebrum | PE150 | D300-1 | 104,963,972 | 100,261,238 | 95.52 | 15.74 | 15.04 | 95.52 | 90.58 | 93.61 |
|  |  |  |  | D300-2 | 81,608,148 | 75,602,064 | 92.64 | 12.24 | 11.34 | 92.64 | 91.57 | 93.86 |
|  |  |  |  | D300-3 | 91,806,682 | 86,935,346 | 94.69 | 13.77 | 13.04 | 94.69 | 90.86 | 93.57 |
| 1 year old | Female | Cerebrum | PE150 | Y1-1 | 131,252,228 | 128,769,822 | 98.11 | 19.69 | 19.32 | 98.11 | 91.77 | 91.96 |
|  |  |  |  | Y1-2 | 126,119,556 | 123,954,976 | 98.28 | 18.92 | 18.59 | 98.28 | 92.21 | 91.87 |
|  |  |  |  | Y1-3 | 135,846,274 | 133,532,462 | 98.30 | 20.38 | 20.03 | 98.30 | 92.7 | 94.40 |
| 3 years old | Female | Cerebrum | PE150 | Y3-1 | 135,100,514 | 132,760,866 | 98.27 | 21.99 | 21.60 | 98.24 | 92.59 | 94.24 |
|  |  |  |  | Y3-2 | 118,997,098 | 116,997,530 | 98.32 | 17.85 | 17.55 | 98.32 | 92.73 | 93.84 |
| 5 years old | Female | Cerebrum | PE150 | Y5-1 | 120,787,644 | 118,638,414 | 98.22 | 21.65 | 21.25 | 98.15 | 92.29 | 95.34 |
|  |  |  |  | Y5-2 | 151,817,914 | 149,057,422 | 98.18 | 22.77 | 22.36 | 98.18 | 92.33 | 95.26 |

**Table S2.** Number of mRNA and lncRNA (including exonic, intronic and intergenic lncRNA) transcripts distributed on **c**hromosome identified in chicken cerebrum

| **Number of Chromosome** | **Length of Chromosome** | **mRNAs** | **lncRNAs** | **intergenic lncRNA** | **intronic lncRNA** | **exonic lncRNA** |
| --- | --- | --- | --- | --- | --- | --- |
| 1 | 195,276,750 | 1687 | 6194 | 3210 | 1233 | 1189 |
| 2 | 148,809,762 | 1087 | 4504 | 2553 | 830 | 697 |
| 3 | 110,447,801 | 1012 | 3992 | 2133 | 749 | 794 |
| 4 | 90,216,835 | 890 | 3572 | 1781 | 653 | 720 |
| 5 | 59,580,361 | 801 | 2532 | 1390 | 523 | 484 |
| 6 | 34,951,654 | 403 | 1199 | 706 | 231 | 193 |
| 7 | 36,245,040 | 420 | 1395 | 783 | 325 | 239 |
| 8 | 28,767,244 | 429 | 1201 | 551 | 300 | 280 |
| 9 | 23,441,680 | 365 | 1273 | 698 | 200 | 292 |
| 10 | 19,911,089 | 366 | 890 | 428 | 176 | 248 |
| 11 | 19,401,079 | 310 | 778 | 412 | 162 | 145 |
| 12 | 19,897,011 | 301 | 785 | 435 | 175 | 172 |
| 13 | 17,760,035 | 277 | 774 | 384 | 146 | 227 |
| 14 | 15,161,805 | 342 | 671 | 294 | 137 | 206 |
| 15 | 12,656,803 | 318 | 551 | 229 | 100 | 205 |
| 16 | 535,270 | 24 | 126 | 17 | 69 | 40 |
| 17 | 10,454,150 | 264 | 454 | 209 | 120 | 122 |
| 18 | 11,219,875 | 268 | 390 | 158 | 98 | 129 |
| 19 | 9,983,394 | 274 | 434 | 145 | 118 | 143 |
| 20 | 14,302,601 | 283 | 602 | 286 | 123 | 174 |
| 21 | 6,802,778 | 213 | 297 | 114 | 71 | 102 |
| 22 | 4,081,097 | 101 | 195 | 113 | 28 | 47 |
| 23 | 5,723,239 | 211 | 304 | 160 | 32 | 105 |
| 24 | 6,323,281 | 148 | 271 | 157 | 40 | 74 |
| 25 | 2,191,139 | 140 | 181 | 58 | 58 | 65 |
| 26 | 5,329,985 | 186 | 301 | 157 | 57 | 87 |
| 27 | 5,209,285 | 198 | 338 | 122 | 120 | 92 |
| 28 | 4,742,627 | 242 | 269 | 143 | 41 | 85 |
| 29 | 0 | 0 | 0 | 0 | 0 | 0 |
| 30 | 0 | 4 | 1 | 0 | 0 | 1 |
| 31 | 0 | 0 | 0 | 0 | 0 | 0 |
| 32 | 1,028 | 12 | 4 | 2 | 1 | 1 |
| 33 | 0 | 66 | 97 | 59 | 13 | 25 |
| Z | 82,363,669 | 587 | 1786 | 1030 | 324 | 329 |
| W | 1,248,174 | 25 | 66 | 2 | 40 | 24 |

**Table S3.** Primer sequences for qPCR

| **Genes/lncRNAs** | **Primers (5'->3')** | **Tm(℃)** | **Product length (bp)** |
| --- | --- | --- | --- |
| BASP1-F  BASP1-R | CCAAGGCCTCAGGTCACAAT  TCCCACGTGGCATTCCTAAC | 59.96  60.04 | 142 |
| GAP43-F  GAP43-R | CGGATGCTTCCAAATCTGCG  GAGAGAGGAGGGGTGTGGAT | 59.97  60.03 | 133 |
| TTR-F  TTR-R | CAACCTCTCCTGCTTTCGCT  GCTACGTTAGCTGCAGGACT | 60.32  59.83 | 109 |
| TMSB15B-F  TMSB15B-R | CGGTTCCGTCGTTGTGAATG  CTTTGTCTGGGCACAAAGCG | 59.84  60.32 | 165 |
| CACNA2D1-F  CACNA2D1-R | TGGCTGAATGGACCCTCAGA  GCCAGCATACCTTTTTGGGC | 60.55  60.11 | 152 |
| FGF13-F  FGF13-R | TGGGTTGGCTGCTGATTAGG  ATGCTGGGCAGAGTAGGGTA | 60.03  60.03 | 157 |
| SHH-F  SHH-R | AGCGAGGATGGGCTTTATGG  ATCTCAATGAGCAGCTCGGG | 59.89  59.89 | 130 |
| CAMK2A-F  CAMK2A-R | GTAGTGCAGAGAAACGGGCT  GTGGGACAGAGTCTGGTGTG | 60.04  59.97 | 143 |
| GRIN1-F  GRIN1-R | ATGGCTCTCACAAGCAGTCC  TTACCTGGCGGAGAGGATGA | 60.04  60.03 | 167 |
| PLP1-F  PLP1-R | CTCTCGGGGTGACATTTGCT  CGTTAGCACGAGACAAGGGT | 60.04  60.04 | 133 |
| TU38242-F  TU38242-R | CAGACGTTTCCCAAAGCACG  ACAAGAGGCTCACAGCTTCC | 60.04  59.96 | 104 |
| TU99158-F  TU99158-R | TGGTCAAGGCGTAGAGGGTA  ATGGGGTTTGCTGGCATGTA | 59.96  59.96 | 194 |
| TU26292-F  TU26292-R | TCCAGGACCCAAATACCCCT  AGCAGAGCCCCTTAACATGG | 59.88  59.74 | 121 |
| TU45029-F  TU45029-R | ACGATTTCAGACCGGTGCAT  AAACACAACACTGCTTCGCC | 60.04  59.90 | 164 |
| TU166039-F  TU166039-R | CCTGAAAGGAAGGGTGGATGT  CCCTCCCCGAATTCAATGAC | 59.64  58.32 | 158 |
| TU56811-F  TU56811-R | AAGCCTTGGTGGACTAGAGC  TAGTAGCTTTGCAGAGGATTGT | 59.38  57.17 | 196 |
| TU6367-F  TU6367-R | AGACTGCTGGTGTCTGAAGC  AACTGGGAGCCCGGTAAAAG | 59.97  59.96 | 133 |
| TU178751-F  TU178751-R | GCCCACTGCAGAGGTTTAGT  ATGGGTGTCTGTGCCATGTT | 59.96  59.89 | 145 |
| TU167089-F  TU167089-R | CGACAGCCTCATTCGTCCG  CGGATCCGACACCTTCCTCT | 60.87  61.04 | 212 |
| TU50759-F  TU50759-R | CGTTGGCAAAGGGGTTTCTG  ACGTGGACCATACCTTGCAC | 59.97  60.32 | 182 |
| β-actin-F  β-actin-R | TTGCACATACCGGAGCCATT  TGACCGGCGGGGTTTATATC | 60.03  59.61 | 102 |


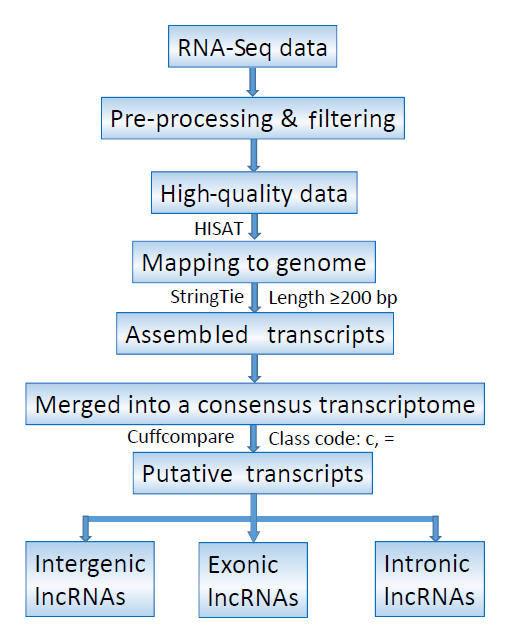


**Figure S1**. Pipeline of lncRNAs identification


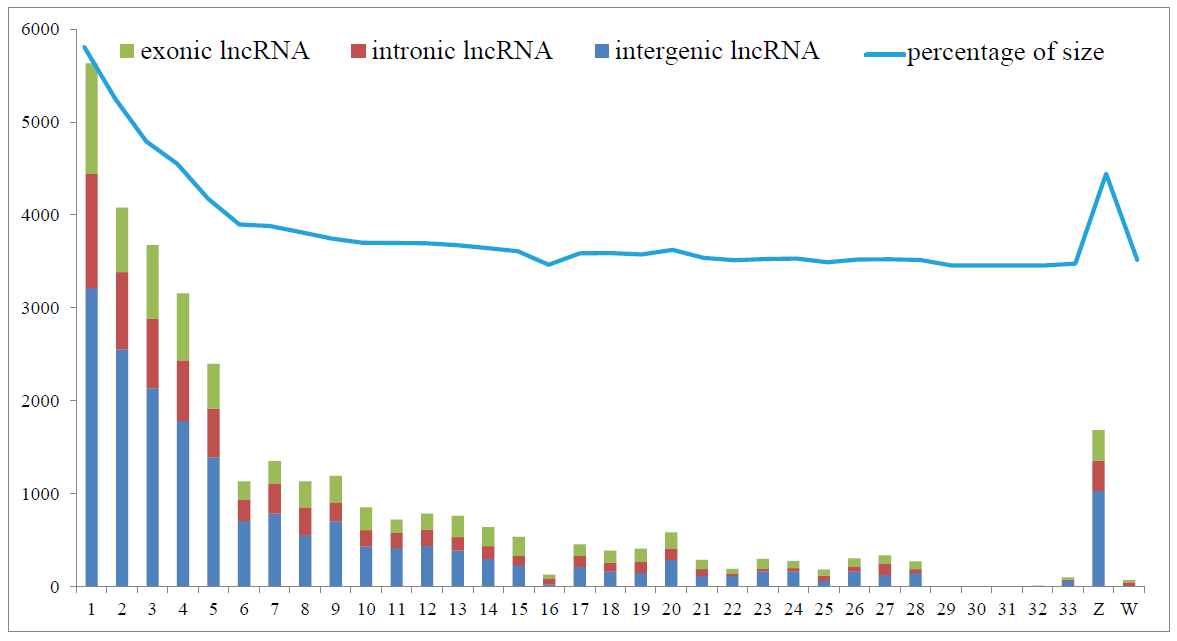


**Figure S2**. Chromosome distribution of 3 subgroups of lncRNAs, blue trend line represents the ratio of chromosome size to genome size. The Pearson correlation of chromosomal size and exonic lncRNA, intronic lncRNA and intergenic lncRNA were 0.0960, 0.9789 and 0.9874, respectively.


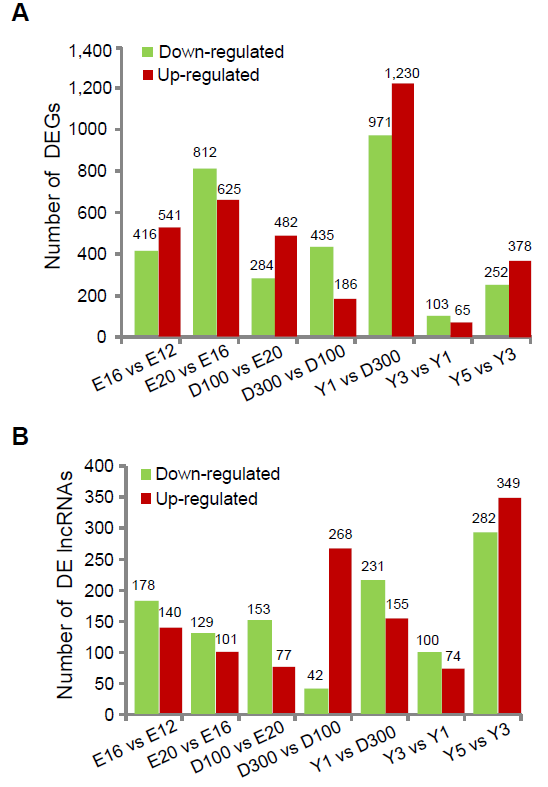


**Figure S3**. Age-dependent mRNAs (A) and lncRNAs (B). Age-dependent genes in each stage were identified through comparing 2 adjacent time points, genes and transcripts with the FC ≥ 2 or ≤ 0.5 plus the *P*-value ≤ 0.05 were identified as age-dependent genes.
